# Plasma-derived exosomal miR-15a-5p as a promising diagnostic biomarker for early detection of endometrial carcinoma

**DOI:** 10.1186/s12943-021-01352-4

**Published:** 2021-03-29

**Authors:** Lanyun Zhou, Wei Wang, Fenfen Wang, Siqi Yang, Jiaqi Hu, Bingjian Lu, Zimin Pan, Yu Ma, Mengyue Zheng, Liyuan Zhou, Shufeng Lei, Penghong Song, Pengyuan Liu, Weiguo Lu, Yan Lu

**Affiliations:** 1 Department of Gynecologic Oncology, Center for Uterine Cancer Diagnosis & Therapy Research of Zhejiang Province, Women’s Reproductive Health Key Laboratory of Zhejiang Province, Women’s Hospital and Institute of Translational Medicine, Zhejiang University School of Medicine, Hangzhou, 310006 Zhejiang China; 2 Department of Clinical Laboratory, Tongde Hospital of Zhejiang Province, Hangzhou, 310006 Zhejiang China; 3 Department of Respiratory Medicine, Sir Run Run Shaw Hospital and Institute of Translational Medicine, Zhejiang University School of Medicine, Hangzhou, 310016 Zhejiang China; 4 Center for Genetic Epidemiology and Genomics, School of Public Health, Soochow University, Suzhou, 215123 Jiangsu China; 5 Department of Surgery, First Affiliated Hospital, Zhejiang University School of Medicine, Hangzhou, 310013 Zhejiang China; 6 Cancer center, Zhejiang University, Hangzhou, 310013 Zhejiang China

**Keywords:** Endometrial cancer, Liquid biopsy, Plasma-derived exosomal miRNA, ddPCR, Cancer diagnosis, Early detection

## Abstract

Endometrial cancer (EC) is a major cause of death among gynecologic malignancies. To improve early detection of EC in patients, we carried out a large plasma-derived exosomal microRNA (miRNA) studies for diagnostic biomarker discovery in EC. Small RNA sequencing was performed to identify candidate exosomal miRNAs as diagnostic biomarkers in 56 plasma samples from healthy subjects and EC patients. These miRNA candidates were further validated in 202 independent plasma samples by droplet digital PCR (ddPCR), 32 pairs of endometrial tumors and adjacent normal tissues by quantitative real-time PCR (qRT-PCR), and matched plasma samples of 12 patients before and after surgery by ddPCR. miR-15a-5p, miR-106b-5p, and miR107 were significantly upregulated in exomes isolated from plasma samples of EC patients compared with healthy subjects. Particularly, miR-15a-5p alone yielded an AUC value of 0.813 to distinguish EC patients with stage I from healthy subjects. The integration of miR-15a-5p and serum tumor markers (CEA and CA125) achieved a higher AUC value of 0.899. There was also a close connection between miR-15a-5p and clinical manifestations in EC patients. Its exosomal expression was not only associated with the depth of muscular infiltration and aggressiveness of EC, but also correlated with levels of reproductive hormones such as TTE and DHEAS. Collectively, plasma-derived exosomal miR-15a-5p is a promising and effective diagnostic biomarker for the early detection of endometrial cancer.

**Supplementary Information:**

The online version contains supplementary material available at 10.1186/s12943-021-01352-4.

## Main text

Endometrial cancer (EC) is the second highest incidence of gynecologic cancer [1]. Patients have to undergo uterine apoxesis for accurate EC diagnosis, since there are no effective biomarkers [2]. Exosomes originate from the endosome, and then fuse with the plasma membrane under the traction of molecular motors, and are released to the extracellular environment [3, 4]. Exosomes are detected in body fluids such as plasma, urine, and amniotic fluid [5]. Exosomes encapsulate biomolecules such as proteins and miRNAs, maintain their integrity in the circulation, and transfer them to recipient cells [4]. MiRNA is the most abundant type in the RNA cargo of exosomes [6, 7], and exosomal miRNAs (exomiRs) are usually tumor-specific [8]. ExomiRs have received increasing attention in precision medicine, due to their non-invasiveness, and high accessibility and stability [2, 9]. Recent studies have shown that exomiRs have the potential to be efficient biomarkers for the screening, diagnosis, and monitoring of cancers [10–13]. However, exomiRs as biomarkers have not yet been reported in EC.

To improve early detection of EC patients, we carried out a large plasma-derived exosomal miRNA study for biomarker discovery in EC (Supplementary Methods). Candidates were identified by miRNA sequencing in plasma samples from healthy controls (HC) vs. EC patients, and were further validated in independent plasma samples and endometrial tumor tissues (Table S1 and Figure S1). Plasma-derived exosomal miR-15a-5p was identified as a promising diagnostic biomarker for early detection of endometrial cancer.

### Identification of exomiRs for EC diagnosis

Exosomes were isolated from plasma of EC patients and age-matched HC subjects. The previously reported method to identify the shape and size of exosomes [5] was used with CD81, TSG101 and GM130 as positive or negative markers, respectively. Indeed, the fraction isolated from plasma was enriched in exosomes (Figure S2).

**Fig. 1:**
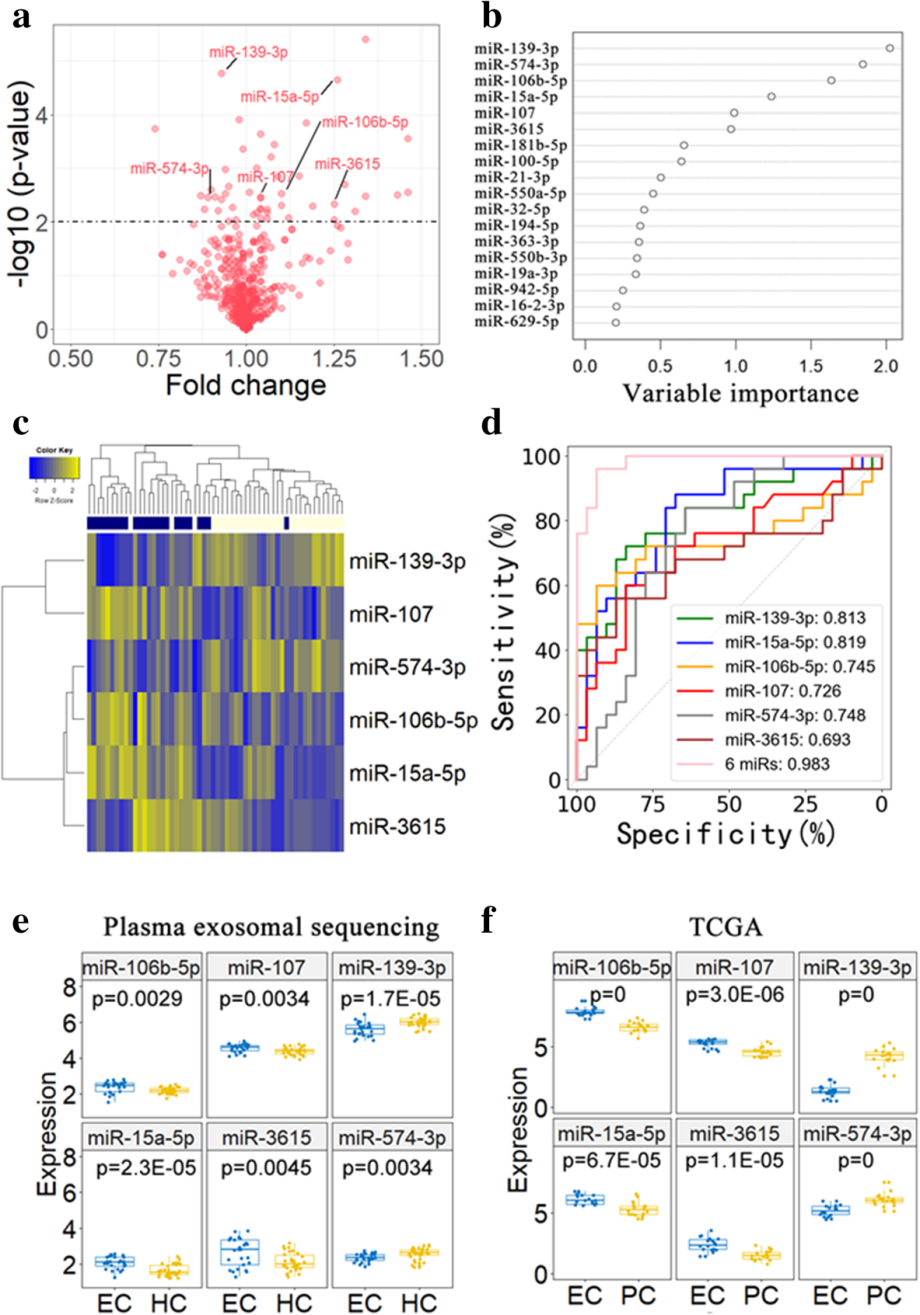
Dysregulated miRNAs in plasma exosomes of EC patients. **a** Volcano plot displaying differentially expressed miRNAs between plasma exosome of EC and HC. Small RNA sequencing was performed in plasma exosomes of 25 EC patients and 31 HC controls. There were 49 differentially expressed with *p*-value < 0.01 (i.e., above the dotted line). **b** Feature vectors forming the best panel to discriminate two different groups were determined by the random forest algorithm. Six miRNAs (miR-106b-5p, miR-107, miR-15a-5p, miR-139-3p, miR-3615, and miR-574-3p) that indicated higher variable importance in the random forest tree were identified as candidate biomarkers. **c** Hierarchical clustering analysis of 6 candidate miRNAs roughly divided plasma samples into two distinct groups (EC vs HC). **d** ROC curves to evaluate the sensitivity and specificity of 6 candidate miRNAs to discriminate EC and HC subjects. **e** The expression levels of 6 candidate miRNAs in plasma exosomes of EC (*n* = 25) and HC (*n* = 31) subjects. **f** The expression levels of 6 candidate miRNAs in EC tumor tissues and matched para-carcinoma tissues from TCGA (*n* = 18). HC: healthy controls; PC, para-carcinoma tissues

The miRNA sequencing was then performed in plasma-derived exosomes from 25 EC and 31 HC subjects. On average, approximately 50 million reads were generated in each library, and 384 exomiRs were detected in each sample (Table S2). Forty-nine miRNAs were differentially expressed between HC and EC groups (*p* < 0.01) (Fig. 1a and Table S3). Eighteen of them also differentially expressed between tumor and adjacent normal tissues in The Cancer Genome Atlas (TCGA) EC samples [14] (Table S4 and Figure S3). Next, a set of exomiRs from these 18 miRNAs were selected as a best panel to distinguish EC from HC subjects using random forest algorithm (Fig. 1b). Clustering analysis showed that these samples were largely divided into two distinct groups (EC vs HC) by these six exomiRs (Fig. 1c). The AUC for each exomiR ranged from 0.693 to 0.819 with a mean of 0.757. The AUC of the combined six exomiRs achieved 0.983 (Fig. 1d). miR-106b-5p, miR-107, miR-15a-5p, and miR-3615 were significantly up-regulated, while miR-139-3p and miR-574-3p were significantly down-regulated in plasma-derived exosomes of EC compared with HC (Fig. 1e). Consistent trends in expression of these exomiRs were observed in tumor tissues from TCGA EC patients (Fig. 1f).

### Validation of diagnostic exomiRs by ddPCR in independent plasma samples

**Fig. 2:**
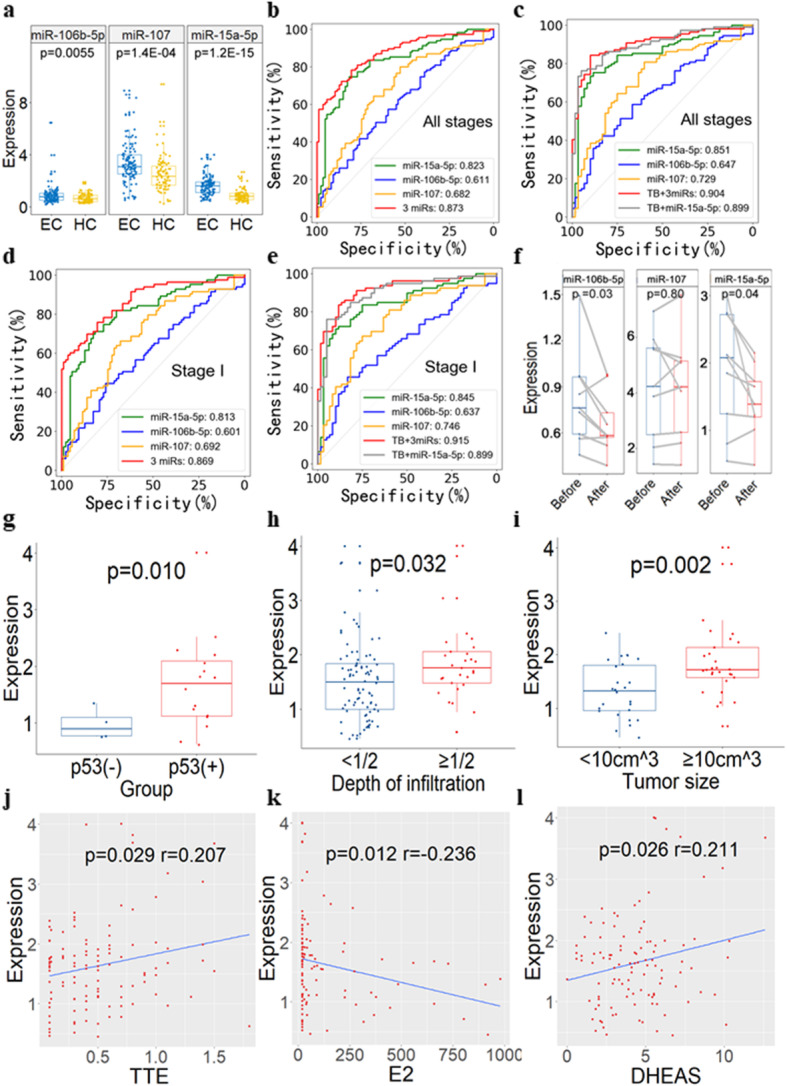
ddPCR validation of plasma exosmal miR-15a-5p, miR-106b-5p and miR-107 as diagnostic markers in 202 independent plasma samples. **a** The expression level of exosomal miR-15a-5p, miR-106b-5p, and miR-107 measured by ddPCR in independent validation samples. **b** ROC curves to validate the discrimination efficiency (for all EC with different stages vs HC) of exosomal miR-15a-5p, miR-106b-5p, miR-107, and their combinations (AUC = 0.832). *n* = 202. **c** ROC curves to validate the discrimination efficiency (for all EC with different stages vs HC) of exosomal miR-15a-5p, miR-106b-5p, miR-107, tumor biomarkers (TB), and their combinations (AUC = 0.885). CEA and CA125 were analyzed in TB analysis. *n* = 169. **d** ROC curves to validate the discrimination efficiency (for stage I EC vs HC) of exosomal miR-15a-5p, miR-106b-5p, miR-107, and their combinations (AUC = 0.815). Only EC patients with stage I in validation samples were analyzed for ROC curves. *n* = 170. **e** ROC curves to validate the discrimination efficiency (for stage I EC vs HC) of exosomal miR-15a-5p, miR-106b-5p, miR-107, TB (i.e., CEA and CA125), and their combinations (AUC = 0.875). *n* = 139. **f** The expression level of plasma-derived exosomal miRNAs from the same EC patients (*n* = 12) before and after surgery quantified by ddPCR. **g-l** The relationship between exosomal miR-15a-5p and clinical manifestations. Plasma-derived exosomal miR-15a-5p is differentially expressed between EC patients with p53 positive and negative staining (**g**), EC patients with depth of muscular infiltration < 1/2 and ≥ 1/2 (**h**), and EC patients with tumor size < 10 cm^3^ and ≥ 10 cm^3^ (**i**). Exosomal miR-15a-5p is positively correlated with TTE (**j**) and DHEAS (**k**), while is negatively correlated with E2 (**l**). TTE: testosterone; DHEAS: dehydroepiandrosterone sulfate; E2: estradiol

Next, we applied droplet digital PCR (ddPCR) to verify these six exomiRs in an independent validation cohort including 115 EC and 87 HC plasma samples. Two stable high-abundance miRNAs (let-7b-5p and miR-26a-5p) were selected as endogenous references, due to their high consistency in expression across all samples (Figure S4). The miR-106b-5p, miR-107, and miR-15a-5p were consistently upregulated in plasma-derived exosomes from EC compared with HC (Fig. 2a). Their upregulation was further verified in 32 pairs of endometrial tumor tissues and adjacent normal tissues using qRT-PCR (Figure S5). While the other three exomiRs were not pursued because of no significant expression changes between EC and HC groups in the validation set or failure of primer design in ddPCR.

The average AUC of these three verified exomiRs was 0.705, ranging from 0.611 to 0.823, in the independent plasma samples. The combination of these exomiRs yielded a much higher AUC than tumor biomarkers (TB, including CEA and CA125) alone (0.873 vs. 0.736) (Figs. 2b and S6). Integration of these exomiRs into routine TB test models significantly improved the diagnostic performance, yielding an AUC of 0.904 (Fig. 2c). To evaluate the efficiency of these exomiRs in the early detection of EC, we specifically analyzed stage I EC patients in the independent plasma samples. Interestingly, the three exomiRs performed reasonably well with an average AUC of 0.702, ranging from 0.601 to 0.813. The AUC of the combined three exomiRs achieved 0.869 (Fig. 2d), while the AUC of TB alone is 0.760 (Figure S6). Integrating these exomiRs into routine TB test models yielded an AUC of 0.915 (Fig. 2e). Particularly, miR-15a-5p yielded an AUC values of 0.823 and 0.813 to distinguish between EC and HC subjects, for all EC patients and stage I EC patients only, respectively. The integration of miR-15a-5p and TB could achieve a higher AUC of 0.899 (Fig. 2b-e).

We also assessed the expression changes of these diagnostic exomiRs after surgery by comparing plasma samples from same patients before and after surgery (*n* = 12). As expected, the expression of miR-15a-5p and miR-106b-5p tended to decrease in EC patients after tumor resection surgery (Fig. 2f), which may be due to the massive reduction of exosomes secreted by tumor cells. Furthermore, there was no significant difference in the exosomal expression of miR-15a-5p, miR-106b-5p, and miR-107 between types I and II of EC patients (*p* > 0.05) (Figure S7).

Taken together, we have identified and validated three plasma-derived exomiRs (miR-106b-5p, miR-107, and miR-15a-5p), which can be served as potential diagnostic biomarkers for both early- and late-stage EC patients.

### Pathway enrichment analysis of diagnostic exomiRs

KEGG pathway enrichment analysis for target genes of the three diagnostic miRNAs were performed to investigate their potential functions involvement in EC (Figure S8). Among the 20 significant pathways (FDR < 0.05), most of them are cancer-related, such as TGF-beta, Hippo, MAPK, p53, FoxO, Wnt, mTOR, and ErbB signaling pathways. The other pathways are related to fatty acid metabolism and biosynthesis, whereas prolactin signaling, oocyte meiosis, and endocytosis pathways are known to be associated with exosome biogenesis and release [14]. These results suggested that these miRNAs can not only serve as diagnostic biomarkers, but also are potentially involved in various steps in EC carcinogenesis and progression.

### Exosomal miR-15a-5p associated with clinicopathologic characteristics

We assessed the relationship between three diagnostic exomiRs and clinicopathologic characteristics in EC patients (Fig. 2g-i). The plasma-derived exosomal miR-15a-5p expression was significantly higher in patients with p53 positive than p53 negative staining (*p* = 0.010). The exosomal miR-15a-5p level increased with the increase of muscular infiltration depth in EC patients (*p* = 0.032). Patients with large tumor had higher exosomal miR-15a-5p expression compared with small tumor (*p* = 0.002). These results implies that exosomal miR-15a-5p expression is strongly predictive of the aggressiveness and p53 mutation status of EC tumors.

Exosomal miR-15a-5p was also associated with testosterone (TTE), dehydroepiandrosterone sulfate (DHEAS), and estradiol (E2) (*p* < 0.05). However, its expression was not associated with normal menstrual cycles (Figure S9). Given a positive association of elevated circulating levels of TTE and DHEAS with EC risk [15], exosomal miR-15a-5p may also be a valuable clinical indicator for EC patients.

### Tissue specificity of miR-15a-5p expression

Finally, we analyzed miR-15a-5p expression in other cancer types, including cervical, breast, ovarian and lung cancer. The miR-15a-5p expression is much more abundant (7–19 times) in EC tumor tissues than that in the other cancer types (Figure S10A). Compared with adjacent tissues, the miR-15a-5p expression was increased to more than 7 times in EC tumor tissues, while the miR-15a-5p was either downregulated in tumor tissues or showed small difference between tumor and adjacent tissues or normal controls of the other cancer types (Figure S10B).

## Conclusions

Our study identified plasma-derived exosomal miR-15a-5p as a valuable diagnostic biomarker for the early detection of EC. Compared with uterine apoxesis, blood extraction is more convenient and carries less risk of vaginal/uterine cervix infection. It has the potential to be incorporated into routine blood examinations for screening endometrial cancer in the general population. Further validation of miR-15a-5p in large sample sizes is warranted before the clinical use as a diagnostic biomarker. Functional investigation of these diagnostic exomiRs will help reveal the mechanisms that underlie the occurrence and development of EC.

## Supplementary Information

**Additional file 1.**

## Data Availability

All data generated during this study are included in this published article and its supplementary files. The sequencing data were deposited in the Genome Sequence Archive (GSA) under accession HRA000737 (http://bigd.big.ac.cn/gsa-human).

## Abbreviations

AUC: Area under receiver operating characteristics curve
CA125: Cancer antigen 125
CEA: Carcinoembryonic antigen
ddPCR: Droplet digital PCR
DHEAS: Dehydroepiandrosterone sulfate
E2: Estradiol
EC: Endometrial cancer
exomiRs: Exosomal miRNAs
FDR: False discover rate
FSH: Follicle stimulating hormone
HC: Healthy controls
LH: Luteinizing hormone
miRNA: microRNA
NTA: Nanoparticle tracking analysis
P: Progesterone
PRL: Prolactin
qRT-PCR: Quantitative real-time PCR
RPM: Reads Per Million mapped reads
TB: Tumor biomarker
TCGA: The Cancer Genome Atlas
TTE: Testosterone
