# Supplementary material for: Plasma-derived exosomal miR-15a-5p as a promising diagnostic biomarker for early detection of endometrial carcinoma
Source: Mol Cancer. 2021 Mar 29;20:57. doi: 10.1186/s12943-021-01352-4 (PMC8006369; doi:10.1186/s12943-021-01352-4)
Supplement: Supplementary file 1 — Additional file 1. [file 12943_2021_1352_MOESM1_ESM.pdf]

Plasma-derived exosomal miR-15a-5p as a promising diagnostic biomarker for early detection of endometrial carcinoma

Lanyun Zhou, Wei Wang, Fenfen Wang, Siqi Yang, Jiaqi Hu, Bingjian Lu, Zimin Pan, Yu Ma, Mengyue Zheng, Liyuan Zhou, Shufeng Lei, Penghong Song, Pengyuan Liu, Weiguo Lu, Yan Lu

SUPPLEMENTARY MATERIALS AND METHODS

INDEX

Supplementary Methods……..…………………………………………..……..….page 2

Supplementary Tables..………………………..………..…...………...…...page 6

Supplementary Figures..………………………… ………………....…...…..…page 12

Supplementary References..……………………… ..……………....…..……….....page 22

Supplementary Methods

Clinical samples

The peripheral blood samples from 140 EC patients who had not been treated with chemotherapy and radiotherapy before surgery, and 118 age-matched HC subjects were collected in the study (**Table S1**). The collected blood samples were centrifuged at 3,000 ×g for 10 min at 4°C to harvest plasma supernatant within 4 hours. The plasma samples were then centrifuged at 16,000 ×g for 10 min at 4°C and then stored at -80°C until use. In the discovery stage, 25 EC patients and 31 HC were used for exosomal miRNA sequencing, while the other samples were used for validation. Additional paired tumor and adjacent normal tissues from 32 EC patients were obtained by surgery without any other treatment. Fresh tissues were collected, snap-frozen in liquid nitrogen, and stored at -80°C. Clinical information, e.g., tumor stage and size, TP53 status, serum tumor markers CEA and CA125, depth of infiltration, body mass index (BMI) and reproductive hormone levels such as testosterone (TTE), estradiol (E2), and dehydroepiandrosterone sulfate (DHEAS), was extracted from patients' medical records at the time of tissue collection. Hematoxylin and eosin (H&E) -stained slides were reviewed by a gynecologic pathologist to confirm the diagnosis.

The method of measuring the depth of muscular layer infiltration is as follows: First, hematoxylin-eosin (HE) slices were made from the tumor. Then, the pathologist observed the tumor cell infiltration under a microscope, made a mark and measured it with a ruler. Finally, the pathologist calculated the depth of muscular layer infiltration (i.e., the proportion of tumor cell infiltration in the entire muscle layer). According to the FIGO, the muscular invasion depth of EC is less than 1/2 in stage IA, and greater than or equal to 1/2 in stage IB. After stage I, the tumor is not limited to the uterine body.

This study was reviewed and approved by the Ethnics Committees of Women's Hospital of Zhejiang University (Hangzhou, China; ID:20170142). The study was conducted in accordance with the International Ethical Guidelines for Biomedical Research Involving Human Subjects (CIOMS). All samples have been collected and utilized following strict human subjects protection guidelines, written informed consent and IRB review of protocols.

Isolation of plasma exosomes and exosomal miRNAs

Plasma samples were centrifuged for 15 min at 3,000 × g at 4℃ to remove possible residual cell debris after thawing the stored samples, and then 250 μl of the samples were transferred into a clean 1.5mL Eppendorf tube. The supernatants were incubated with pre-warmed thromboplastin D (Thermo Scientific Cat #: 100356, Grand Island, NY) at 37℃ for 15 min, followed by a centrifugation at 10,000 ×g for 5 min at room temperature, and then supernatants were transferred into fresh 1.5mL tubes for exosome isolation.

Exosomes were isolated using an ExoQuick Exosome Precipitation Solution (SBI Cat #:100356EXOQ20A-1, Mountain View, CA) mixture with RNase A (Sigma Cat#: R6513-10MG, St. Louis, MO) at a final concentration of 10 μg/ml. The mixtures were incubated in a 4℃ refrigerator for at least 12 hours. A total of 150 units/ml of murine RNase inhibitor (NEB Cat#: M0314L, Ipswich, MA) were added to the mixtures followed by precipitating the

extracellular vesicles by centrifugation at 1,500 × g at room temperature for 30 min. The resulting exosome pellets were slightly washed and re-suspended in 25 µl sterile PBS.

The exosomal miRNA was isolated by miRNeasy Micro Kit (QIAGEN Cat#: 217084, Valencia, CA) following the manufacturer's protocol. The quantity of exosomal miRNAs were measured by the Qubit^{TM}microRNA Assay Kit (Invitrogen Cat# Q32881) on the Qubit® 2.0 Fluorometer.

MiRNA library preparation and next generation sequencing

Twenty-five EC and 31 HC samples were randomly selected for miRNA sequencing from our tissue repository. Sequencing libraries were constructed with 2-10 ng of small RNA from plasma-derived exosomes of these samples using the NEBNext Multiplex Small RNA Library Prep Set (NEB Cat # E7560) following the manufacturer's protocol. The sequencing libraries were purified using the QIAquick PCR Purification Kit (QIAgen Cat #: 28106), fragments ranging from 140 to 160 bp were selected by 3% agarose gel, and the miRNA libraries were eluted in 10 µl 10 mM Tris-HCI, pH 8.5. The size distribution and quantity of sequencing libraries were determined using the DNA 1000 Kit (Agilent Technologies Cat #: 5067-1505) on Agilent 2100 Bioanalyzer. Before RNA sequencing, 24 libraries with different indices were pooled at equal concentration. Pooled libraries were quantified by RT-qPCR using the KAPA Library Quantification Kit (KAPA Biosystems, Wilmington, MA) and were sequenced on an Illumina HiSeq X10 analyzer by Mingma Company (Shanghai, China) (**Table S2**).

MiRNA sequencing data analysis

The adapter sequences were removed from raw reads and low-quality sequences (base quality < 20) at both ends of the reads were further trimmed by Trim Galore (https://www.bioinformatics.babraham.ac.uk/projects/trim_galore/). The trimmed reads were aligned to the human reference genome (GRCH37.p5) using the Burrows-Wheeler Aligner [1], allowing for no mismatch base pairs per read. Reads aligned to miRNAs (miRbase v20) were counted using the bedtools (http://bedtools.readthedocs.io/en/latest). miRNA expression levels were quantified by Reads Per Million (RPM) mapped reads. miRNAs with zero RPM in more than 90% of samples were removed. RPM values were then converted to log2(RPM+1), followed by quantile normalization, and removing batch effect using combat package [2]. In addition, miRNA sequencing data of 18 pairs of EC tumor and matched adjacent normal tissues (**Table S4**) were downloaded from The Cancer Genome Atlas (TCGA) (https://portal.gdc.cancer.gov/). Similarly, reads in TCGA samples were processed and analyzed as described above.

Droplet digital PCR

The expression levels of plasma exosomal miRNAs were measured by ddPCR. Briefly, primers of miRNAs were synthesized by Sangon Biotech (**Table S5**). Reverse transcription of miRNA was performed using miRNA First Strand cDNA Synthesis (Tailing Reaction) (Sangon Biotech Cat#: B532451-0020, Shanghai, China). EvaGreen Supermix (Biorad Cat#: 186-4035) 10µl, forward and reverse primers 0.2µl (10µM), cDNA, and RNase free water were mixed in 20µl solution. The automated Droplet Generator (Bio-Rad) was used for eumulsification, and the ddPCR cycle conditions were 95℃ for 10 min, followed by 40 cycles of a 2-step thermal

profile of 94℃ denaturation for 15 sec, 57℃ annealing for 60 sec, and the followed by a 4℃ hold. After the 96-well plate was loaded on to and read by a QX200 Droplet Reader (Bio-Rad), results were analyzed using QuantaSoft™ Analysis Pro (Bio-Rad). miR-26a-5p and let-7b-5p were selected as internal miRNAs to adjust for loading error since they showed small variability across samples and no significant expression difference between EC and HC groups (**Fig. S4**). The ddPCR uses absolute quantitative principles, so each miRNA expression level was normalized using the following formula: miRNA expression/the average of the internal miRNAs expression.

Total RNA isolation and quantitative real-time PCR

Total RNA was extracted from ~20mg tumor or adjacent normal tissues by TRIzol (Life Technologies Cat#: 15596018) according to the manufacturer's protocol. The quality and quantity of RNA were measured with an ND-1000 spectrophotometer (NanoDrop Technologies, Rockland, DE, USA). q-RTPCR was performed with miRNA First Strand cDNA Synthesis (Sangon Biotech Cat#: B532451-0020) to quantify the expression level of miRNA. The miRNA expression was normalized to U6 and analyzed for relative fold changes from the threshold cycle (Ct) values using the $2^{-\Delta\Delta Ct}$ method.

Electron microscopy

Suspended plasma exosomes in particle-free PBS (0.02 μm filtered) were dropped onto a carbon-coated copper grid (200 mesh), allowed to incubate at room temperature for 5 min, and then the liquid was removed by an absorbent tissue. Uranyl acetate was applied to the grid and left for 10 sec. Next, the residual reagent was removed with filter paper and air dried for 30 min, then the exosome suspension retained was imaged with a transmission electron microscope (TEM) (JEOL USA, Inc.).

Nanoparticle tracking analysis (NTA)

Plasma exosome samples were diluted in 500μl particle-free PBS (0.02 μm filtered) and analyzed by NanoSight NS500 instruments (Malvern Instruments, Amesbury, UK). The focal length and camera level of the instrument were adjusted according to the manufacturer's software manual. Five videos of 45-sec duration were taken, and particle movement was analyzed using NTA software (NTA 3.4 software).

Western blot

Isolated plasma exomes were suspended in lysis buffer (50 mM Tris-HCl PH 8.0, 1% SDS, 1 mM EDTA, 5 mM DTT, 10 mM PMSF, 1 mM NaF, 1 mM Na3VO4, and protease inhibitor cocktail), and then denatured in boiling water for 10 min. The cellular lysates were centrifuged at 13,000 rpm for 30 min. The protein concentration was determined by a BCA assay (Thermo Fisher Scientific, Waltham, MA, USA). Equal amount of proteins (40 μg) were loaded into a sodium dodecyl polyacrylamide gel electrophoresis (SDS-PAGE) with 10% gel. The proteins were then transferred onto a polyvinylidene fluoride (PVDF) membrane. The membrane was blocked with 5% skim milk and incubated with the antibodies. The antibodies used included rabbit anti-TSG10 and anti-CD81 (**Table S4**). Immunoreactive bands were developed by enhanced chemiluminescence reaction (Pierce) following standard protocols.

Statistical analysis

Two-sample t test with permutation was used to examine the difference of miRNAs between two groups. Random forest, an ensemble machine learning method, was used to choose feature vectors that formed the best panel to discriminate two different groups, implemented in the R package “randomForest”. The results of hierarchical clustering using miRNA expression profiles were displayed in the heatmap, which was created using the R function “heatmap” with dist method 'manhattan' and hclust method 'average'. Area under receiver operating characteristics curve (AUC) was used to measure the efficiency of miRNA for distinguishing between HC and EC groups. The AUC value was calculated using the function “auc” in the R packages “pROC.” MiRNA target genes prediction and KEGG pathway enrichment analysis were performed with DIANA-miRPath (v3) [3]. False discover rate (FDR) < 0.05 from the enrichment analysis was set as the threshold indicating a statistically significant pathway. MiRNA-gene network was used to identify the relationship between candidate miRNAs and their target genes through Cytoscape software (Cytoscape_v3.6.1). All statistical analyses were all performed in the R Statistical Package (v3.5.1) (www.r-project.org).

Table S1 Demographic and clinicopathologic characteristics of the study subjects.

	Discovery set		Validation set	
**Characteristics***	**EC**	**HC**	**EC**	**HC**
**Age**				
<40			4	5
40-60	18	18	84	61
>60	7	13	27	21
**Type**				
Type I	18		-51	
Type II	4		11	
Mixed			48	
Unknown	3		5	
**Stage**				
I	17		82	
II	5		15	
III	2		17	
IV	1			
Unknown			1	
**Depth of infiltration**				
<1/2			86	
⩾1/2			29	
**Tumor size**				
<10cm^3			26	
⩾10cm^3			29	
Unknown			60	
**p53 status**				
Positive			16	
Negative			4	
Unknown			95	
**CEA**			1.95 (n=109)	2.42 (n=86)
**CA125**			37.02 (n=110)	14.61 (n=60)
**LH**			26.27 (n=110)	
**FSH**			49.07 (n=110)	
**TTE**			0.50 (n=110)	
**E2**			124.44 (n=110)	
**P**			1.46 (n=110)	
**PRL**			13.99 (n=110)	
**DHEAS**			4.39 (n=110)	

*CEA: carcinoembryonic antigen; CA125: cancer antigen 125; LH: luteinizing hormone; FSH: follicle stimulating hormone; TTE: Testosterone; E2: Estradiol; P: progesterone; PRL: prolactin; DHEAS: dehydroepiandrosteronesulfate. These characteristics are expressed as mean and sample size (numbers in parentheses).

Table S2. Quality control metrics of plasma exosomal miRNA sequencing libraries.

Sample ID	Yield (Gb)	# Reads	% of >=Q30 Bases	Mean Quality Score	miRs
HC1	4.74	55,238,935	91.85	36.98	344
HC2	4.29	49,039,012	92.6	37.23	290
HC3	2.94	44,021,401	94.16	37.67	275
HC4	4.55	53,711,131	92.22	37.13	267
HC5	6.26	74,389,173	93.45	37.3	276
HC6	2.43	30,304,485	96.08	37.81	306
HC7	3.53	44,528,485	93.52	37.38	305
HC8	5.69	76,143,752	90.68	36.75	354
HC9	4.45	56,821,544	95.29	37.69	347
HC10	2.61	33,702,728	94.39	37.47	339
HC11	3.57	45,436,935	89.11	36.44	328
HC12	1.19	15,699,809	94.94	37.58	322
HC13	3.39	42,843,446	93.42	37.37	319
HC14	4.2	58,726,953	96.78	38.04	337
HC15	3.26	46,984,718	97.01	38.04	347
HC16	3.9	43,232,656	92.41	37.26	454
HC17	3.8	49,507,139	93.87	37.67	431
HC18	4.29	50,062,914	94.36	37.68	465
HC19	4.89	64,095,547	95.13	37.73	453
HC20	6.27	83,412,591	96.04	37.85	478
HC21	4.32	57,672,733	95.11	37.81	486
HC22	3.73	50,893,145	96.81	38.1	485
HC23	4.69	63,445,264	96.18	37.99	439
HC24	4.24	57,942,778	96.35	38.09	446
HC25	2.14	29,648,931	98.61	38.54	480
HC26	2.47	35,881,795	98.77	38.57	490
HC27	4.84	62,818,347	98.3	38.44	486
HC28	2.88	39,898,033	98.19	38.37	446
HC29	3.05	41,688,839	98.01	38.34	453
HC30	3.01	38,339,601	97.47	38.26	421
HC31	3.43	46,625,533	97.45	38.26	445
EC1	3.15	50,147,710	97.55	38.25	287
EC2	3.29	51,219,497	97.49	38.18	354
EC3	2.71	36,849,671	94.44	37.62	327
EC4	3.16	33,460,848	97.11	37.96	377
EC5	5	66,258,361	93.14	37.36	340
EC6	4.03	41,635,088	87.21	36.17	312
EC7	2.81	36,787,647	91.08	37.01	308
EC8	4.57	55,510,244	92.06	36.99	319
EC9	3.06	40,293,958	94.89	37.63	295
EC10	4.98	68,634,003	96.45	37.99	269
EC11	3.65	44,675,471	96.58	37.95	282
EC12	5.15	71,103,836	96.6	37.85	358
EC13	3.87	56,495,266	96.29	38.01	297
EC14	4.71	66,524,566	96.19	37.92	327
EC15	4.2	58,123,479	96.41	38.08	453

EC16	4.13	55,760,384	96.25	38.03	455
EC17	2.97	40,277,754	96.28	37.97	492
EC18	3.35	45,533,911	96.67	38.07	469
EC19	3.5	48,890,279	98.07	38.41	441
EC20	4.38	62,548,866	99.19	38.61	427
EC21	2.81	37,658,228	97.94	38.28	442
EC22	3.97	53,949,592	97.89	38.27	439
EC23	3.39	47,059,596	98.17	38.32	436
EC24	3.45	48,051,697	97.68	38.27	453
EC25	3.02	40,829,128	97.93	38.32	455

Table S3. Forty-nine miRNAs differentially expressed between endometrial cancer patients and healthy controls (p<0.01).

miRNA	HC_RPM	EC_RPM	Fold change	p-value
miR-503-5p	1.72	1.78	1.03	4.0E-06
miR-139-3p	82.26	48.96	0.60	1.7E-05
miR-15a-5p	6.20	5.28	0.85	2.3E-05
miR-767-5p	139.59	93.69	0.67	1.2E-04
miR-497-5p	22.01	17.02	0.77	1.4E-04
miR-215-5p	3.61	1.60	0.44	1.8E-04
miR-130a-3p	59.28	53.05	0.89	2.3E-04
miR-4732-5p	0.77	1.44	1.87	2.7E-04
miR-486-3p	691.32	1525.63	2.21	3.6E-04
miR-100-5p	12113.92	8955.22	0.74	4.4E-04
miR-486-5p	914.83	2032.30	2.22	6.0E-04
miR-126-5p	533.01	426.73	0.80	9.7E-04
miR-146b-5p	45.34	25.56	0.56	0.0010
miR-16-2-3p	24.08	50.39	2.09	0.0013
miR-219a-5p	32.94	28.97	0.88	0.0014
miR-21-3p	0.90	0.98	1.09	0.0020
miR-375	80.11	49.76	0.62	0.0021
miR-365a-5p	4.09	3.00	0.73	0.0024
miR-181b-5p	3719.01	3285.42	0.88	0.0028
miR-550b-3p	0.31	0.74	2.37	0.0028
miR-106b-5p	13.70	13.41	0.98	0.0029
miR-487a-5p	24.59	14.52	0.59	0.0029
miR-550a-5p	0.35	0.84	2.44	0.0031
miR-197-3p	11.85	6.07	0.51	0.0032
miR-942-5p	0.85	1.39	1.63	0.0033
miR-107	61.08	45.21	0.74	0.0034
miR-574-3p	17.59	10.05	0.57	0.0034
miR-1246	3.05	2.58	0.85	0.0035
miR-363-3p	96.49	91.44	0.95	0.0035
miR-377-5p	27.37	14.96	0.55	0.0037
miR-3615	6.10	11.12	1.82	0.0045
miR-19a-3p	23.32	20.67	0.89	0.0048
miR-1180-3p	3.43	6.97	2.03	0.0050
miR-326	204.45	98.68	0.48	0.0052
miR-499b-3p	49.04	47.47	0.97	0.0056
miR-200b-3p	8.04	3.86	0.48	0.0058
miR-499a-5p	49.05	47.47	0.97	0.0058
miR-194-5p	55.42	57.03	1.03	0.0058
miR-1911-5p	8.66	4.55	0.52	0.0061
miR-3158-5p	0.81	1.77	2.17	0.0062
miR-32-5p	42.78	44.44	1.04	0.0066
miR-222-3p	539.58	447.10	0.83	0.0067
miR-27a-3p	1278.65	1438.77	1.13	0.0072
miR-629-5p	12.95	32.62	2.52	0.0083
miR-451a	6879.75	15311.23	2.23	0.0084
miR-1197	20.17	18.43	0.91	0.0087
miR-885-3p	44.90	29.44	0.66	0.0090
miR-101-5p	0.84	0.50	0.59	0.0091
miR-105-3p	19.87	13.26	0.67	0.0095

Table S4. List of TCGA EC BAM files used in the study.

TCGA EC ID
TCGA - AJ - A2QL - 11A - 11R - A18L - 13
TCGA - AJ - A3NC - 11A - 11R - A22I - 13
TCGA - AJ - A3NE - 11A - 11R - A22I - 13
TCGA - AJ - A3NH - 11A - 11R - A22I - 13
TCGA - AX - A1CF - 11A - 11R - A136 - 13
TCGA - AX - A1CI - 11A - 11R - A136 - 13
TCGA - AX - A1CK - 11A - 11R - A136 - 13
TCGA - AX - A2H8 - 11A - 11R - A17A - 13
TCGA - AX - A2HA - 11A - 11R - A18L - 13
TCGA - AX - A2HC - 11A - 11R - A17A - 13
TCGA - AX - A2HD - 11A - 11R - A17A - 13
TCGA - BG - A2AD - 11A - 11R - A16E - 13
TCGA - BG - A3EW - 11A - 22R - A22I - 13
TCGA - BG - A3PP - 11A - 11R - A22I - 13
TCGA - BK - A4ZD - 11A - 12R - A27R - 13
TCGA - DI - A2QU - 11A - 11R - A18L - 13
TCGA - DI - A2QY - 11A - 11R - A19V - 13
TCGA - E6 - A1M0 - 11A - 11R - A143 - 13
TCGA - AJ - A2QL - 01A - 11R - A18L - 13
TCGA - AJ - A3NC - 01A - 11R - A22I - 13
TCGA - AJ - A3NE - 01A - 11R - A22I - 13
TCGA - AJ - A3NH - 01A - 11R - A22I - 13
TCGA - AX - A1CF - 01A - 11R - A136 - 13
TCGA - AX - A1CI - 01A - 11R - A136 - 13
TCGA - AX - A1CK - 01A - 11R - A136 - 13
TCGA - AX - A2H8 - 01A - 11R - A17A - 13
TCGA - AX - A2HA - 01A - 12R - A18L - 13
TCGA - AX - A2HC - 01A - 11R - A17A - 13
TCGA - AX - A2HD - 01A - 21R - A17A - 13
TCGA - BG - A2AD - 01A - 21R - A16E - 13
TCGA - BG - A3EW - 01A - 11R - A22I - 13
TCGA - BG - A3PP - 01A - 11R - A22I - 13
TCGA - BK - A4ZD - 01A - 11R - A27R - 13
TCGA - DI - A2QU - 01A - 11R - A18L - 13
TCGA - DI - A2QY - 01A - 12R - A19V - 13
TCGA - E6 - A1M0 - 01A - 11R - A143 - 13

Table S5. List of primers and antibodies used in this study.

Antibodies	Company (catalogue number)
GM130	Abcam (ab52649)
TSG101	Abcam (ab125011)
CD81	Abcam (ab109201)
**miRNA**	**primer sequence**
let-7b-5p	CGTGAGGTAGTAGGTTGTGTGGTT
miR-106b-5p	AGGTAAAGTGCTGACAGTGCAGAT
miR-107	AGCAGCATTGTACAGGGCTATCA
miR-139-3p	ATAATGGAGACGCGGCCCTG
miR-15a-5p	CACGTAGCAGCACATAATGGTTTGTG
miR-26a-5p	CCGTTCAAGTAATCCAGGATAGGCT
miR-574-3p	ACACGCTCATGCACACACC

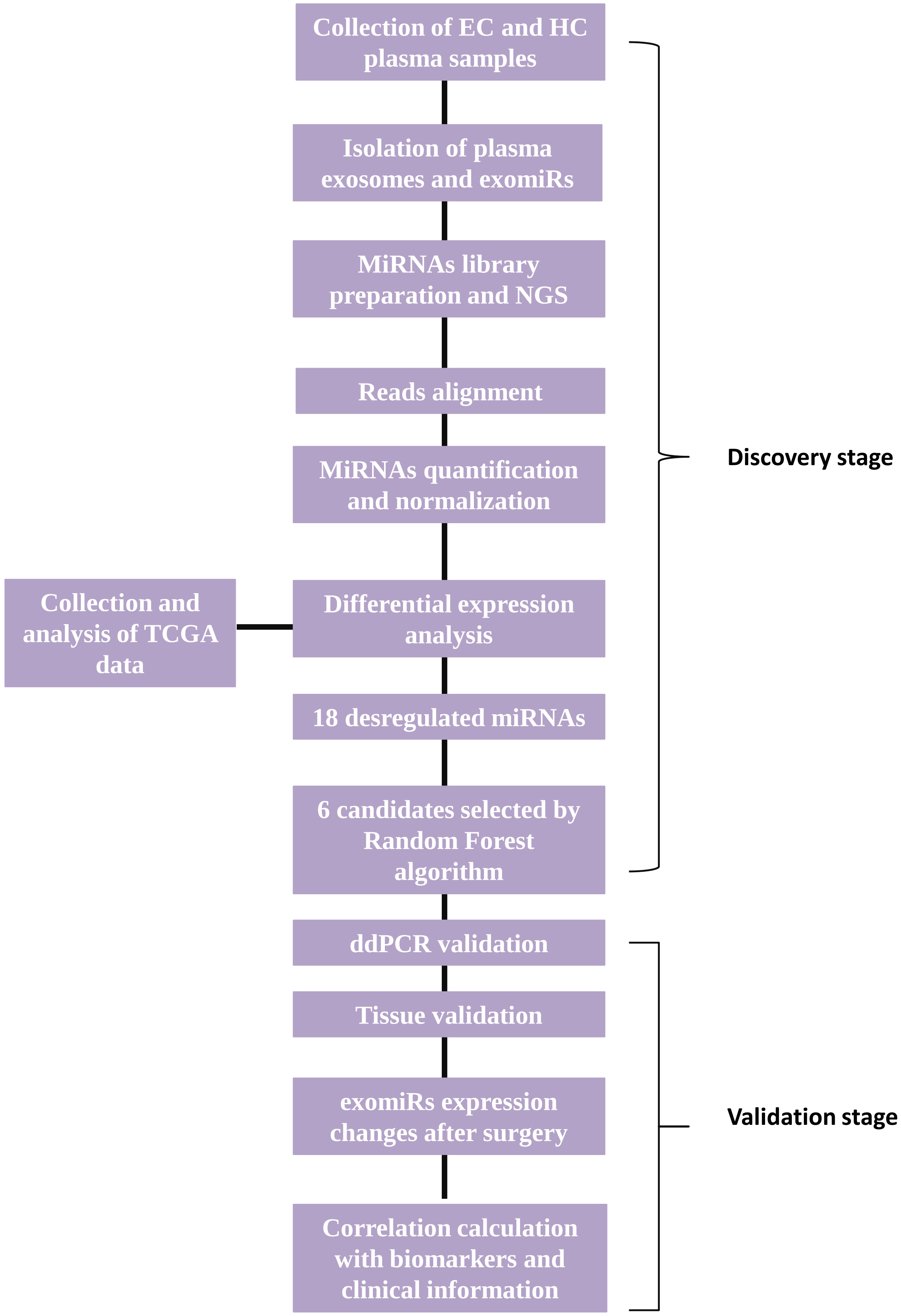

Figure S1. A workflow for discovery and validation of endometrial cancer biomarker.

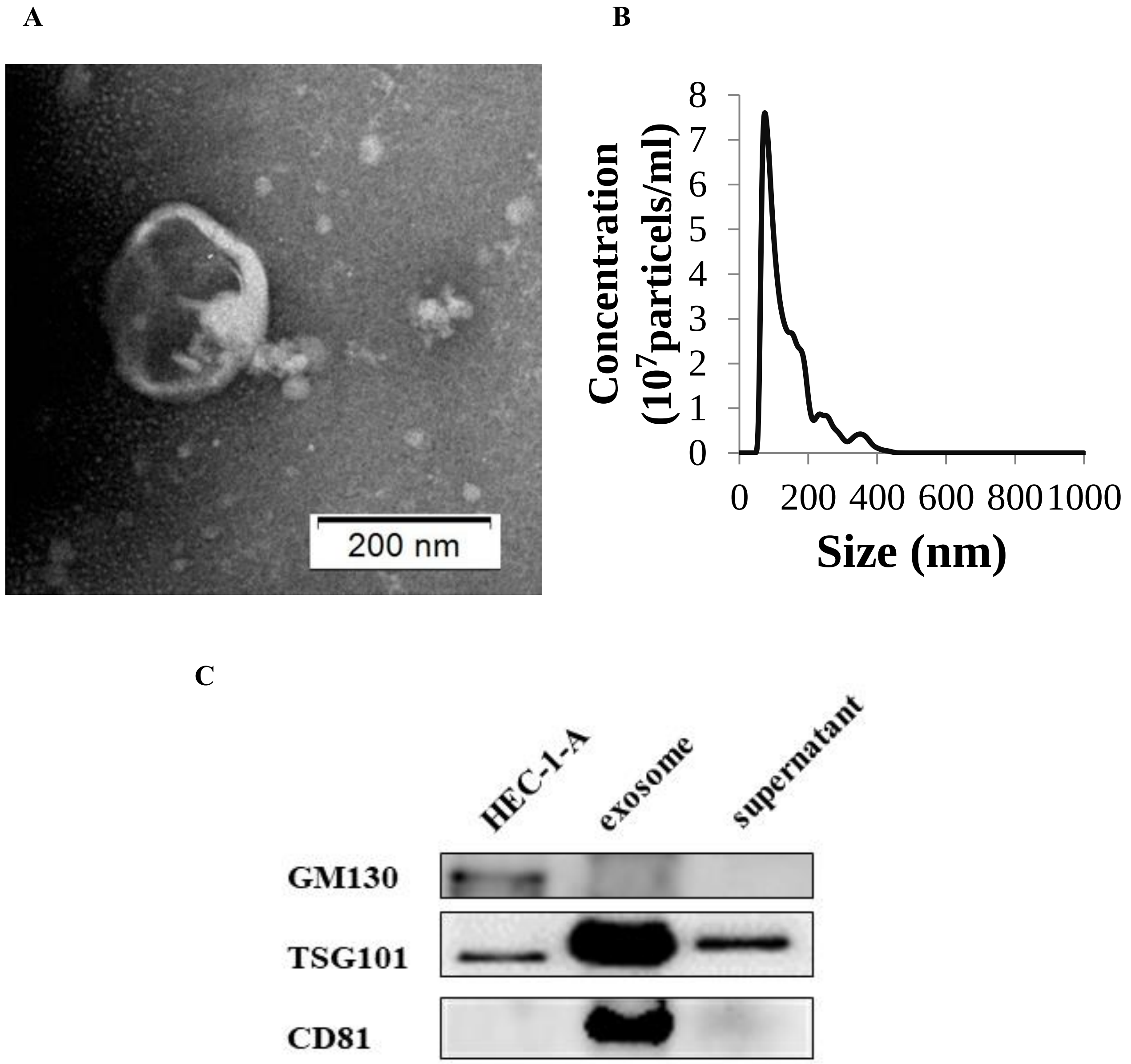

Figure S2. Isolation and characterization of plasma exosomes. (A) Transmission electron microscopy images of plasma exosomes isolated from blood samples. **(B)** Exosome size distribution measured by NanoSight. **(C)** GM130, TSG101 and CD81 in exosomes by Western blot. CD81 and TSG101 (Tumor Susceptibility 101) are marker genes of exosomes, while GM130 (Golgi matrix protein) is a negative marker gene of exosomes.

A

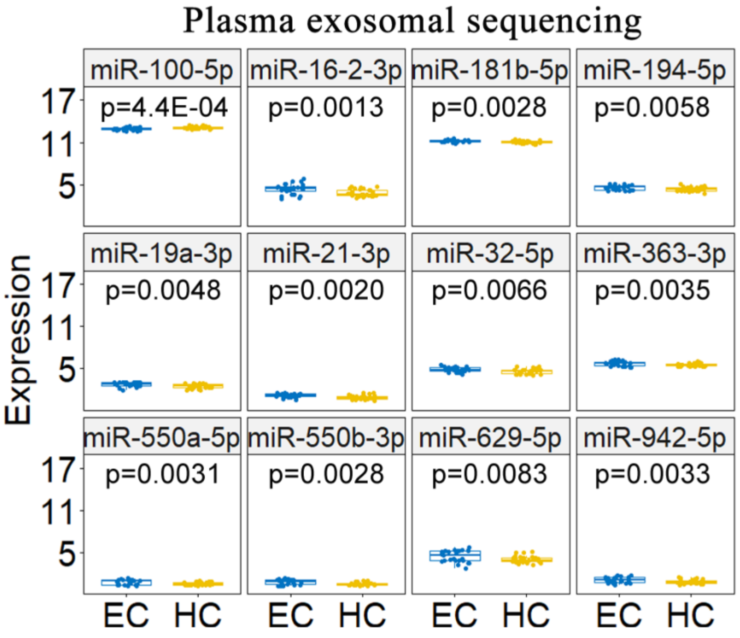

B

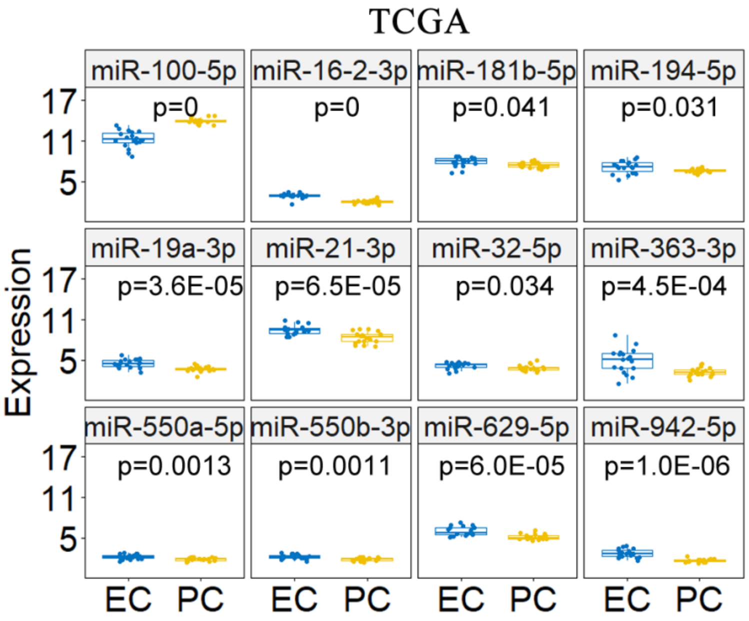

Figure S3. Other 12 miRNAs showing consistent trends in both our plasma exosomal miRNA sequencing (A) and TCGA samples (B). EC: endometrial cancer; HC: healthy controls; PC: paracancerous tissue.

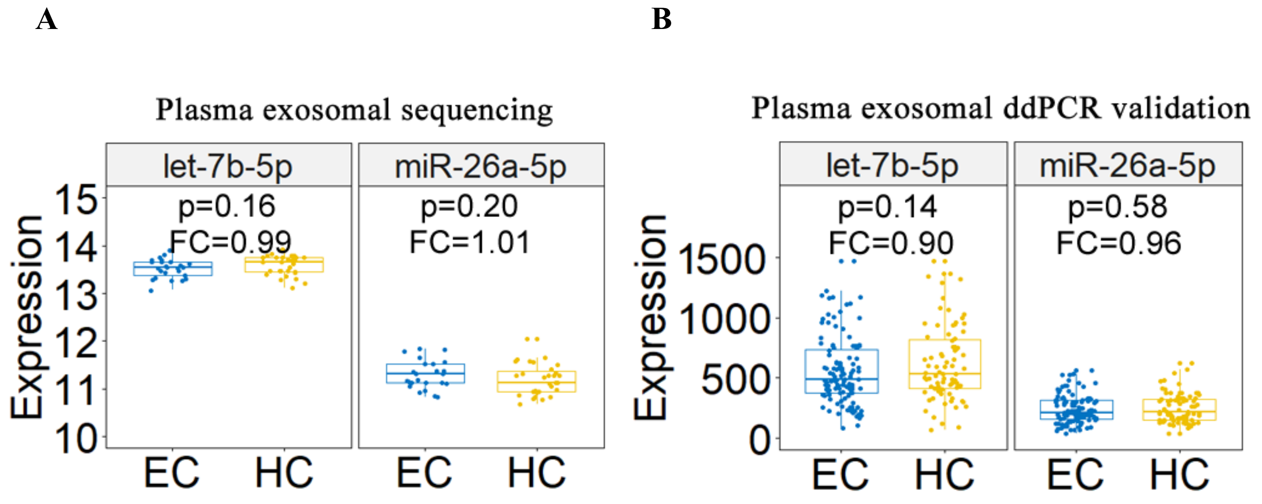

Figure S4. Internal miRNAs for normalized expression of miRNAs measured by ddPCR. (**A**) Expression of let-7b-5p and miR-26a-5p in plasma exosomal miRNA sequencing samples. (**B**) Expression of let-7b-5p and miR-26a-5p in ddPCR validation samples. These two miRNAs showed no expression differences between EC and HC, and had a small overall coefficient of variation among samples. EC: endometrial cancer; HC: healthy controls.

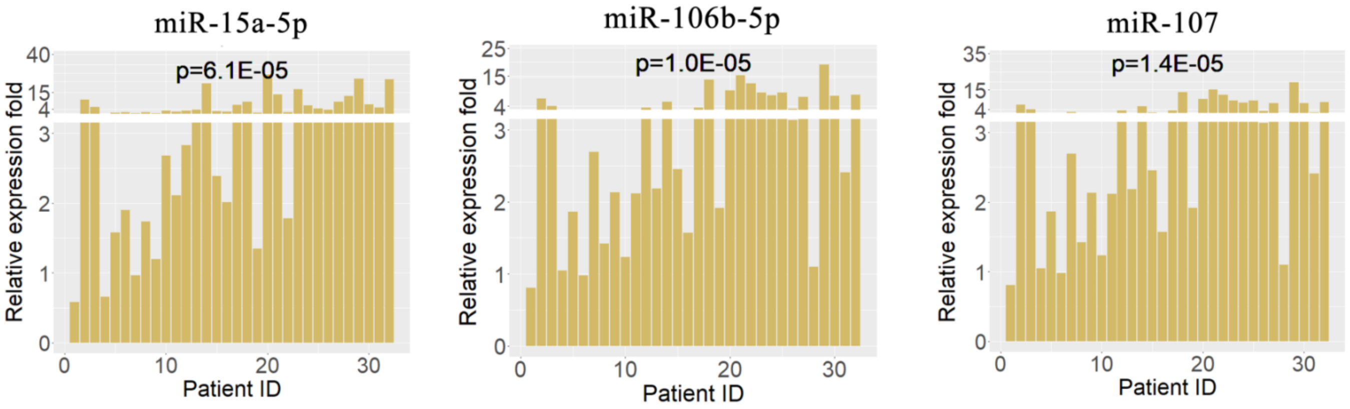

Figure S5. Expression levels of miR-15a-5p, miR-106b-5p and miR-107 validated in endometrial carcinoma tissues and matched para-carcinoma tissues. Relative expression fold = expression of miRNAs in tumor tissue / expression of miRNAs in matched para-carcinoma tissues.

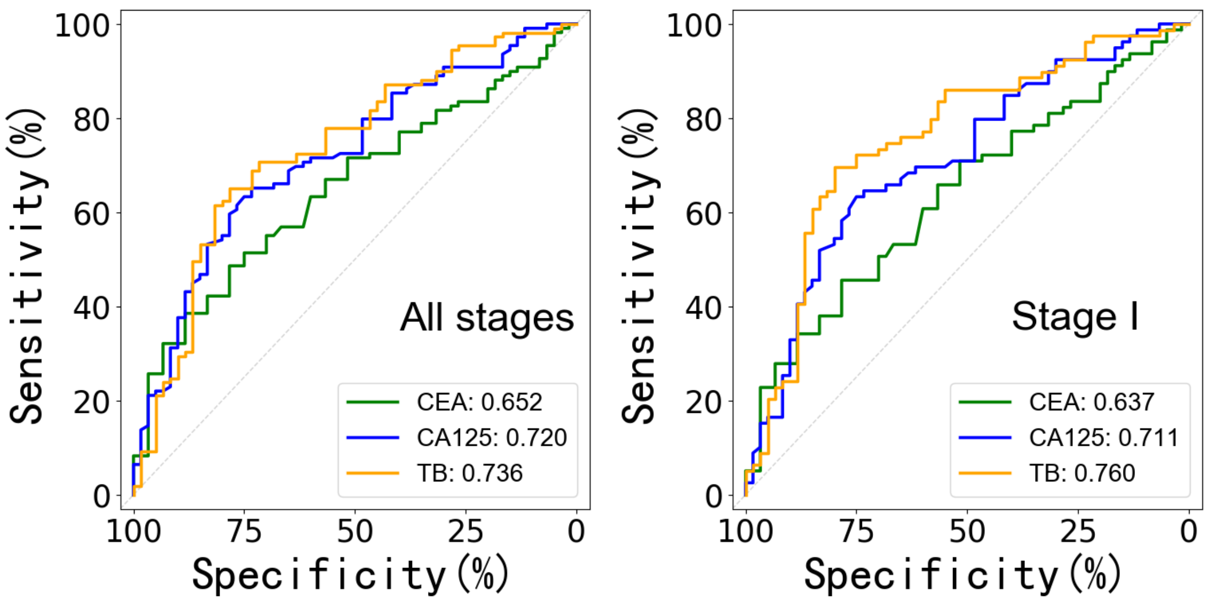

Figure S6. ROC curves of tumor biomarkers to evaluate the sensitivity and specificity of tumor biomarkers to discriminate EC and HC subjects. (**A**) All EC patients and HC. (**B**) stage I ECs and HC. EC: endometrial cancer; HC: healthy controls.

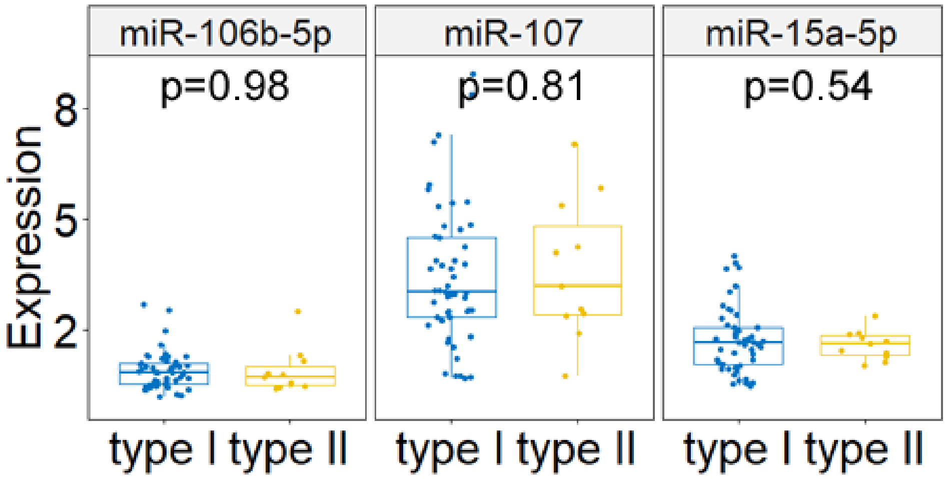

Figure S7. Exosomal expression of three diagnostic miRNAs in types I and II of EC patients. EC patients that were deterministically classified as type I or type II were analyzed. Difference in exosomal miRNA expression between type I and type II was compared using two-sample t test.

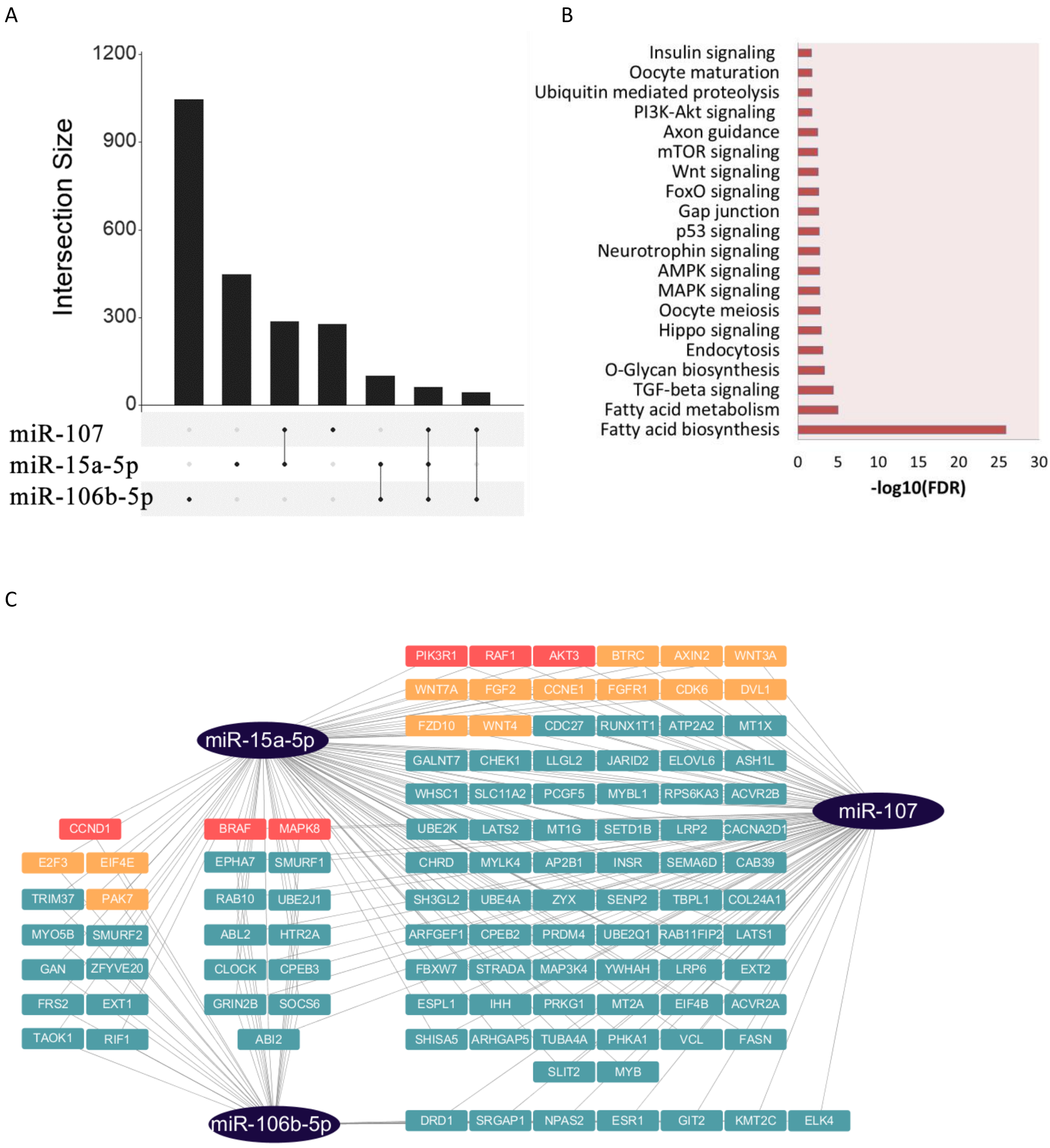

Figure S8. Functional enrichment analysis of target genes of three miRNA candidates. (**A**) Numbers of overlapped target genes of 3 biomarker miRNAs (predicted form microT-CDS in miRPath v3). (**B**) KEGG pathways enrichment analysis of target genes of three biomarker miRNAs. (**C**) MiRNA-gene regulatory network. Green, yellow, and red ovals represent target genes involved in 1-5, 6-10, and >10 pathways, respectively. The top 20 pathways were presented in the figure.

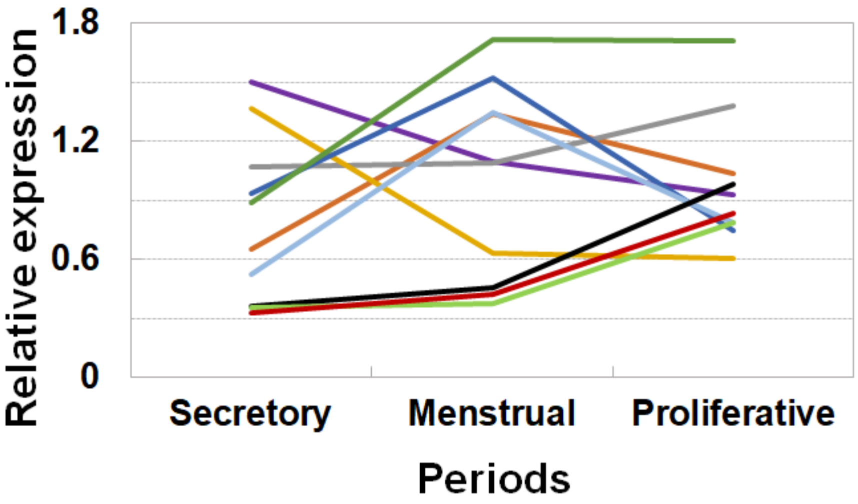

Figure S9. Plasma-derived exosomal expression of miR-15a-5p among menstrual cycles
Plasmas samples were collected from 10 healthy controls (HC); three samples were took from each HC during menstrual, proliferative, and secretory periods. ANOVA was used to compare miR-15a-5p expression among three periods (p=0.384). Two-sample paired t test was used to compare miR-15a-5p expression between any two periods. Secretory-menstrual period: p=0.254; menstrual-proliferative period: p=0.887; and proliferative- secretory period: p=0.296.

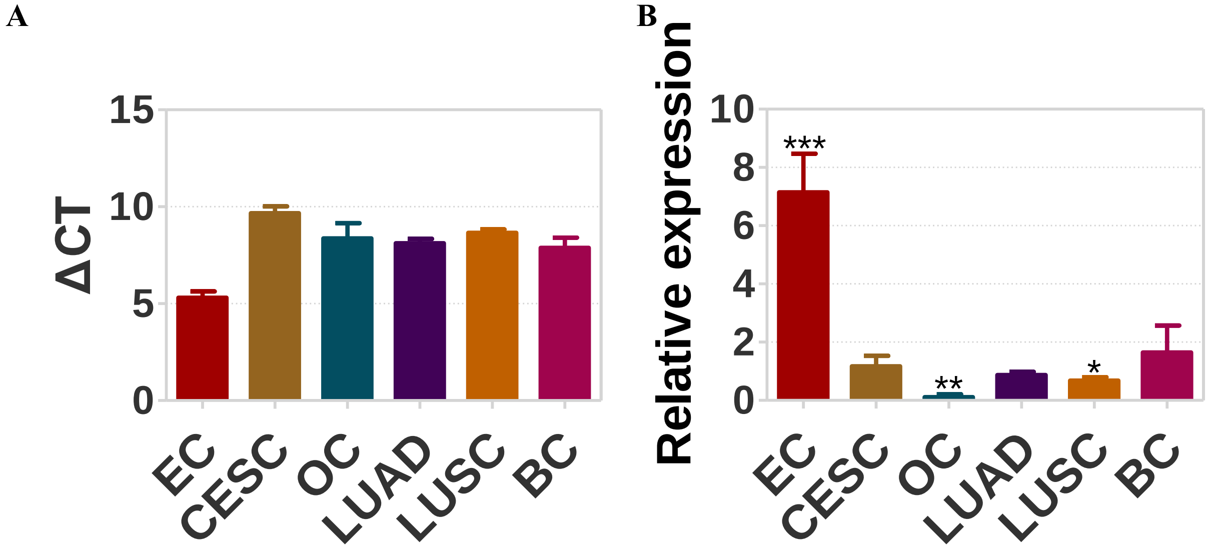

Figure S10. Expression of miR-15a-5p in other cancer types. (**A**) Abundance of miR-15a-5p in tumor tissues of different cancer types. ΔCT = CT of miR-15a-5p – CT of U6. The smaller the ΔCT, the higher the expression of miR-15a-5p in tumor tissues. CT represents cycle threshold value in PCR. (**B**) Relative expression of miR-15a-5p between tumor tissues and adjacent tissues of different cancer types. The miR-15a-5p expression level was quantified by qRT-PCR in tumor tissues and adjacent tissues of endometrial cancer (EC) (n=32), cervical cancer (CC) (n=12), breast cancer (BC) (n=13), ovarian cancer (OV) (n=10), lung adenocarcinoma (LUAD) (n=15), and lung squamous cell carcinoma (LUSC) (n=12). Fallopian tube was used as normal controls in OV. The miRNA expression was normalized to U6. Two sample t test was used to compare miR-15a-5p expression difference between tumor tissues and normal tissues of OV. Two sample paired t test was used to compare miR-15a-5p expression difference between tumor tissues and adjacent tissues in the other cancer types. $^*P < 0.05$, $^{**}P < 0.01$ and $^{***}P < 0.001$.

Supplementary References

1. Li H, Durbin R: **Fast and accurate short read alignment with Burrows-Wheeler transform.** *Bioinformatics* 2009, **25:**1754-1760.
2. Johnson WE, Li C, Rabinovic A: **Adjusting batch effects in microarray expression data using empirical Bayes methods.** *Biostatistics* 2007, **8:**118-127.
3. Vlachos IS, Zagganas K, Paraskevopoulou MD, Georgakilas G, Karagkouni D, Vergoulis T, Dalamagas T, Hatzigeorgiou AG: **DIANA-miRPath v3.0: deciphering microRNA function with experimental support.** *Nucleic Acids Res* 2015, **43:**W460-466.
